# Supplementary material for: The Intestinal Microbiome in Dogs with Chronic Enteropathies and Cobalamin Deficiency or Normocobalaminemia—A Comparative Study
Source: Animals (Basel). 2023 Apr 17;13(8):1378. doi: 10.3390/ani13081378 (PMC10135184; doi:10.3390/ani13081378)
Supplement: Supplementary file 1 [file animals-13-01378-s001.zip › Supplementary_Figures S1 and S2.pdf]

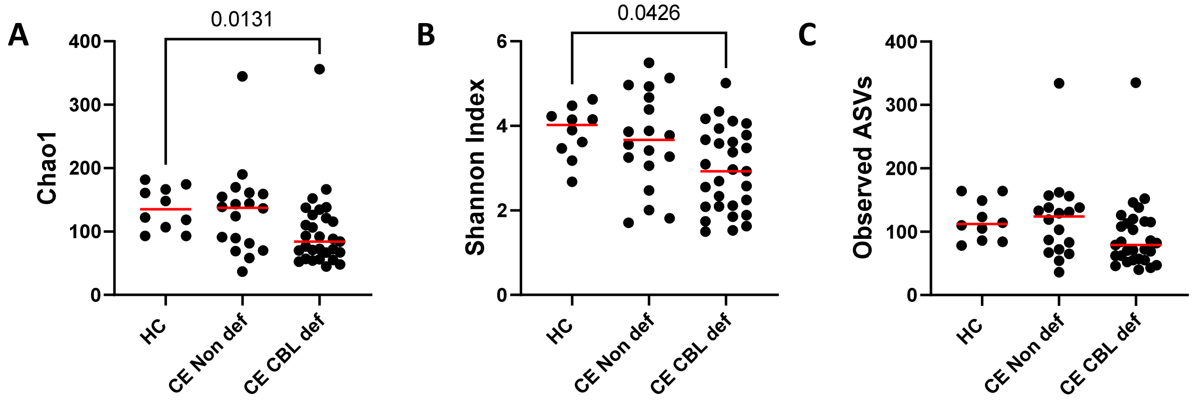

Supplementary Figure S1. *Alpha-diversity scores in dogs with chronic enteropathies (CE) with or without cobalamin (CBL) deficiency at inclusion, compares to healthy controls (HC). (A) Non-parametric richness (Chao1) and (B) Shannon Index, a measure of both richness and evenness were both significantly decreased in dogs with CE and cobalamin deficiency. (C) Observed ASVs, a parametric measure of richness, was not significantly different from healthy controls.*

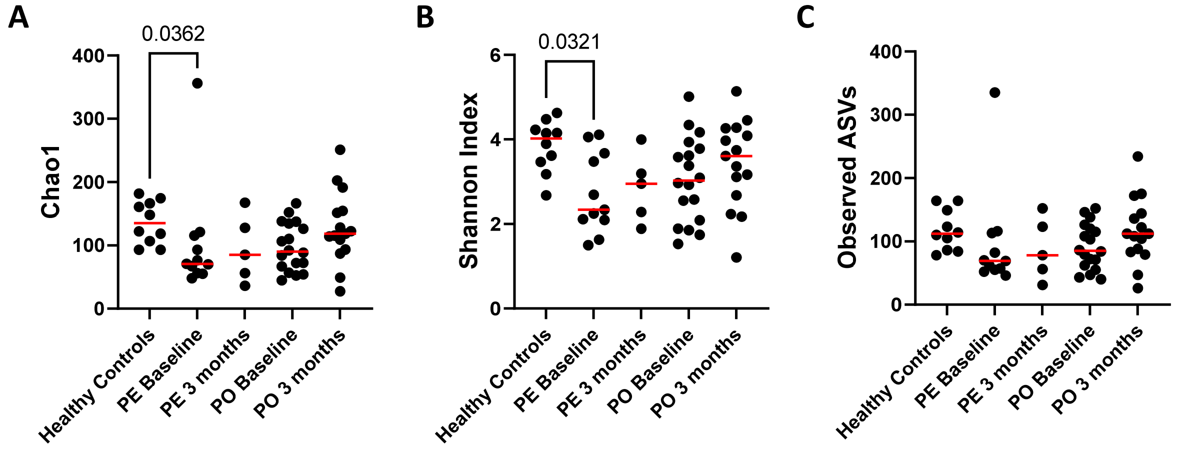

Supplementary Figure S2. *Alpha-diversity scores in dogs with CE and cobalamin deficiency at inclusion and after 3 months of cobalamin supplementation (combined with appropriate therapy on a case-by-case basis), separated by route of administration (oral, PO; or parenteral, PE). (A) Non-parametric richness (Chao1) and (B) Shannon Index, a measure of both richness and evenness were both significantly decreased at baseline in the PE group but not PO at baseline. (C) Observed ASVs, a parametric measure of richness, was not significantly different from healthy controls at baseline or at 3 months.*
